# Early-onset hypertension associated with a CACNA1H variant of uncertain significance: a case report and literature review

**DOI:** 10.1186/s12872-026-05587-1

**Published:** 2026-02-04

**Authors:** Xiang Fang, Ruhui Liu, Jing Zeng

**Affiliations:** 1 Department of General Internal Medicine, West China Second University Hospital, Sichuan University, No.20, Section 3, Ren Min Nan Lu, Chengdu, 610041 Sichuan China; 2 Key Laboratory of Birth Defects and Related Diseases of Women and Children, Sichuan University, Ministry of Education, Chengdu, 610041 Sichuan China

**Keywords:** Familial hyperaldosteronism type IV, Early-onset hypertension, Genetic study, Pathogenic variant, Incomplete penetrance, Plasma aldosterone, Case report

## Abstract

**Background:**

The prevalence of early-onset hypertension is rising annually and is accompanied by progressive target organ damage, contributing to a higher risk of cardiovascular mortality. In patients with early-onset hypertension characterized by refractory hypertension, elevated plasma aldosterone levels, and a family history of hypertension, monogenic hereditary hypertension, such as familial hyperaldosteronism, should be suspected, although this condition is rare.

**Case presentation:**

A 36-year-old male patient with hypertension fails to achieve target blood pressure despite receiving four antihypertensive medications, including a diuretic. The patient exhibited elevated plasma aldosterone levels, while the aldosterone-to-renin ratio and serum potassium levels remain within normal ranges. Further genetic analysis identifies a heterozygous variant of uncertain significance in the *CACNA1H* gene (nucleotide change: c.3988G > A, amino acid change: p.V1330I, chromosomal location: chr16:1260601). This genetic variant has not been previously reported. The *CACNA1H* gene is associated with familial hyperaldosteronism type IV. Sanger sequencing validation and family pedigree analysis were performed, confirming an autosomal dominant inheritance pattern among family members.

**Conclusions:**

For patients with early-onset hypertension characterized by refractory hypertension, elevated plasma aldosterone levels, and a family history of hypertension, monogenic forms of hypertension, such as familial hyperaldosteronism, should be suspected. However, For patients with negative ARR but atypical clinical manifestations of elevated aldosterone levels, exclusive reliance on common biochemical markers, such as the aldosterone-to-renin ratio and serum potassium levels, may lead to misdiagnosis or underdiagnosis. Therefore, in addition to routine biochemical markers, genetic testing should be considered a complementary diagnostic tool for patients with early-onset hypertension and a family history of hypertension.

## Background

Early-onset hypertension is defined as hypertension diagnosed at or before the age of 45 [1], with an increasing prevalence over time. According to the National Health and Nutrition Examination Survey, the prevalence of early-onset hypertension increased from 6.4% in 2001 to 7.8% in 2007 [2]. From 2012 to 2015, early-onset hypertension constituted 14.9% of the total hypertensive population in China [3]. Recent studies have demonstrated that early-onset hypertension is associated with a higher burden of clustered cardiovascular risk factors, more severe target organ damage, and an increased risk of cardiovascular mortality [4–7]. Additionally, early-onset hypertension demonstrates significant familial aggregation [8]. Therefore, a comprehensive understanding of the pathophysiology and genetic underpinnings of early-onset hypertension is crucial for timely diagnosis and effective management, as well as for mitigating its socioeconomic burden.

Hypertension follows two genetic architectures: common polygenic essential hypertension, in which multiple small-effect genes interact with environmental factors to produce a heterogeneous, late-onset phenotype, and rare monogenic hypertension, caused by a single Mendelian mutation with autosomal dominant or recessive transmission [9, 10]. It typically manifests at a young age with refractory hypertension, severe target organ damage, increased cardiovascular mortality, and familial aggregation [10, 11]. To date, 18 types of monogenic hypertension have been identified, including familial hyperaldosteronism, Liddle syndrome and Gordon syndrome. Among these, familial hyperaldosteronism manifests as primary hyperaldosteronism [10–13]. Primary aldosteronism accounts for 5.9% to 22.8% of newly - diagnosed hypertension cases. Most are sporadic, with familial aldosteronism making up less than 9% [12, 13]. Familial hyperaldosteronism, an autosomal dominant subtype, is categorized into types I-IV. Notably, FH-IV is very rare, making up less than 1% of primary aldosteronism [13, 14]. Monogenic hypertension, including familial hyperaldosteronism, represents a distinct subset of secondary hypertension. Its autosomal dominant or recessive inheritance makes it particularly relevant in early-onset hypertension with familial aggregation, often indicating an underlying genetic mutation disrupting blood pressure regulation [11–14]. However, incomplete penetrance or medications may lead to atypical presentations with negative aldosterone-to-renin ratio (ARR) but elevated aldosterone, increasing the risk of misdiagnosis or delayed diagnosis [14]. Here, we report a case of early-onset hypertension associated with a CACNA1H variant of uncertain significance and elevated plasma aldosterone levels despite a normal aldosterone-to-renin ratio. The patient carries a previously unreported genetic variant. By analyzing the genetic and pathophysiological aspects of this case, we aim to improve disease recognition and reduce diagnostic errors.

## Case presentation

**Fig. 1:**
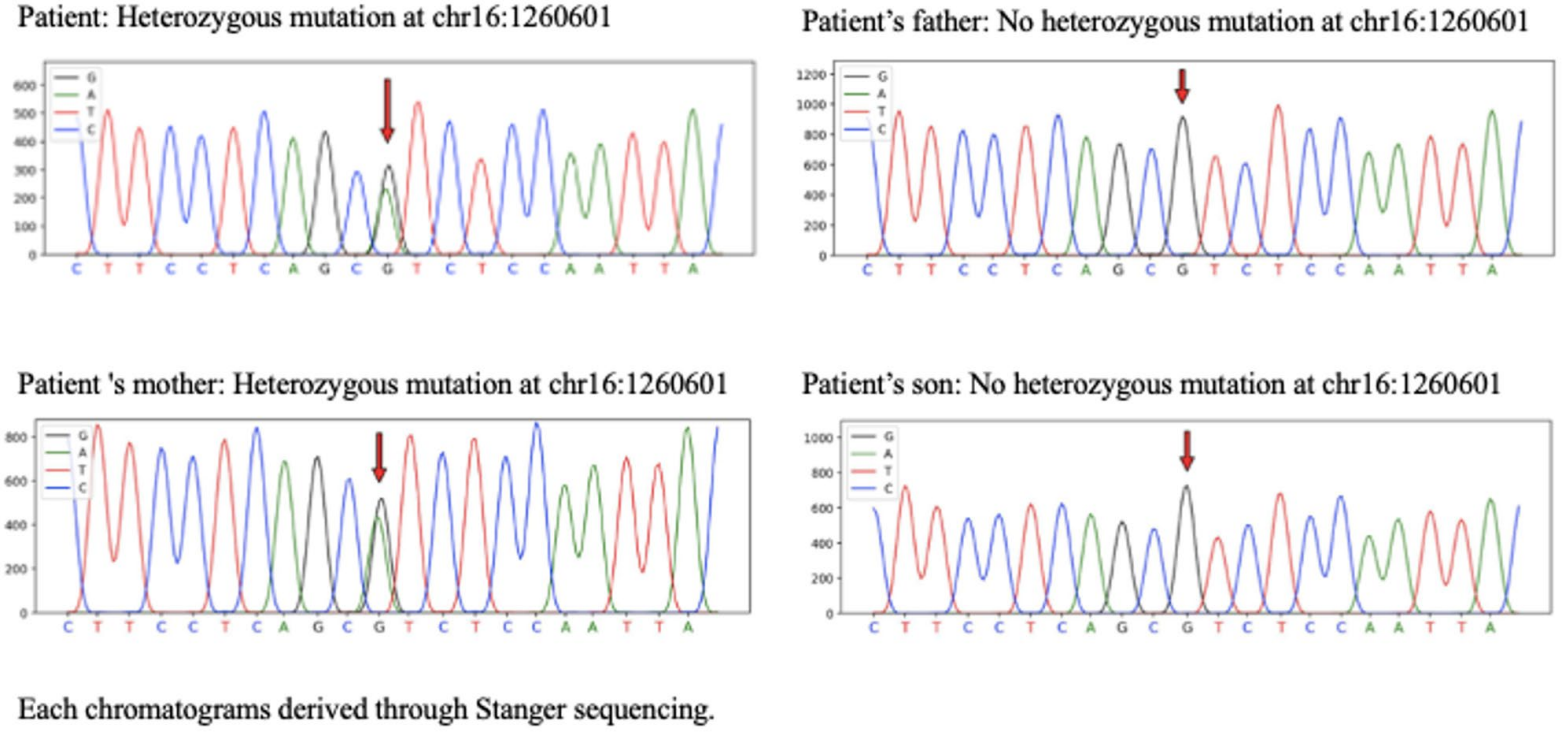
Genetic Testing Results of the Patient and Family Members

**Fig. 2:**
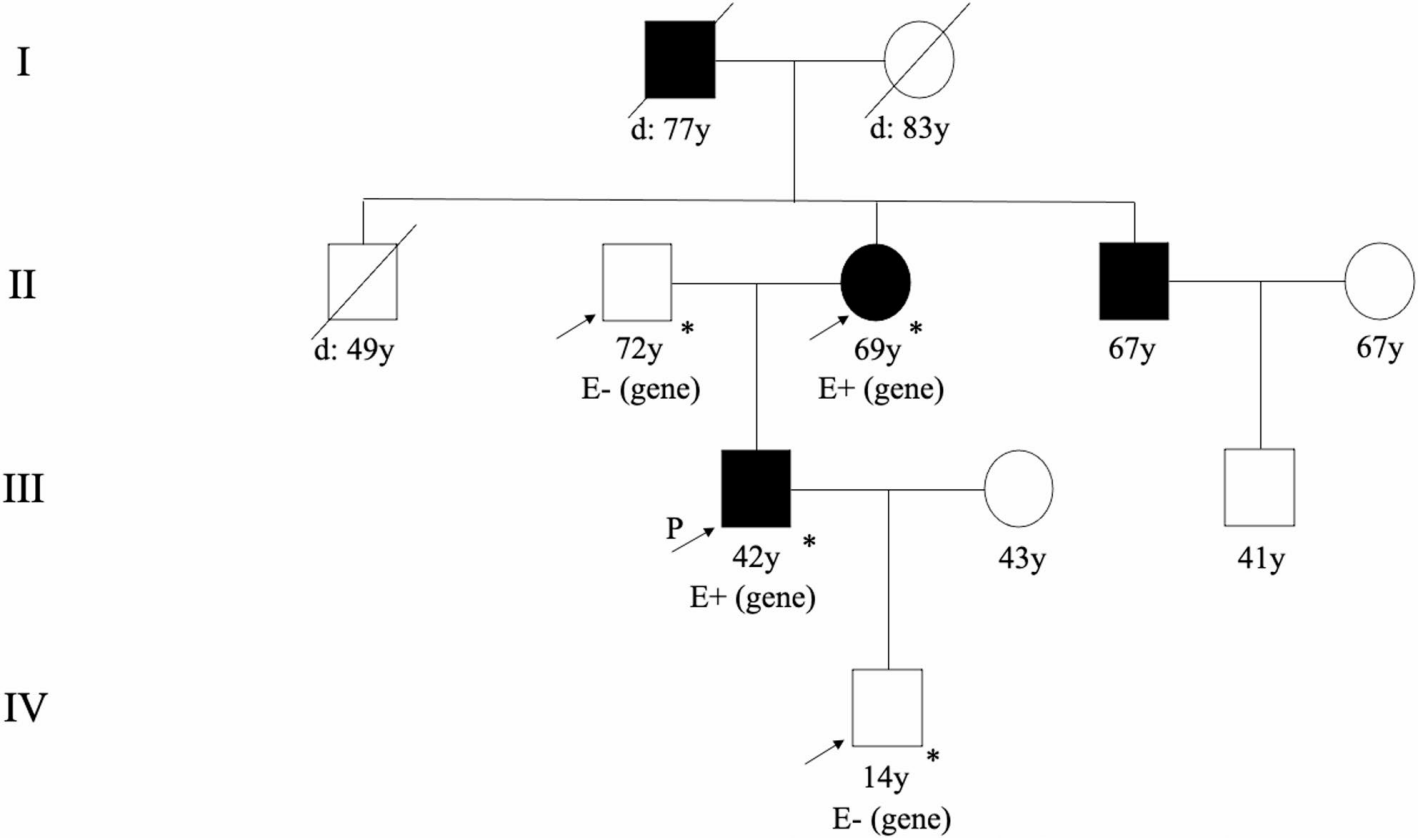
Pedigree Chart of the Patient. y: represents age; d: represents death; a 45-degree slash: represents deceased individuals; *: represents having undergone a certain test; E- (gene): represents the test for the gene is negative; E+ (gene): represents the test for the gene is positive; an arrow: represents having received genetic counseling; P: represents the proband. The proband (patient), the proband’s mother, the proband’s uncle, and the proband’s grandfather all suffer from hypertension

**Fig. 3:**
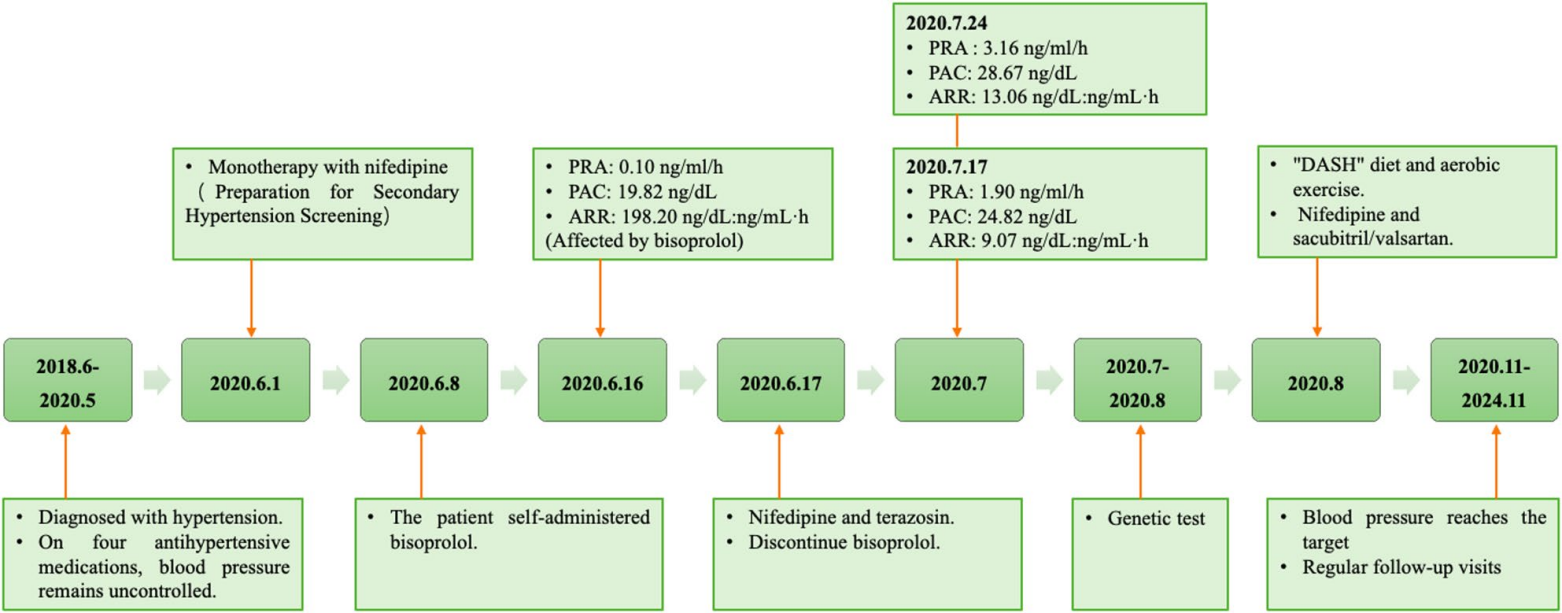
The diagnosis and treatment history of the patient. ARR aldosterone-to-renin ratio; PRA plasma renin activity; PAC plasma aldosterone concentration

A 36-year-old male patient was diagnosed with hypertension in June 2018, with a peak blood pressure of 180/120 mmHg, accompanied by symptoms of snoring. He was diagnosed with type 2 diabetes at the age of 38. His family history revealed early-onset hypertension (diagnosed before age 45) in his mother, maternal uncle, and maternal grandfather, and his mother also has type 2 diabetes. The patient was initially treated with sustained-release nifedipine (30 mg once daily), irbesartan (150 mg once daily), hydrochlorothiazide (25 mg once daily), and metoprolol (47.5 mg once daily), achieving partial blood pressure control within 130–140/90–100 mmHg. On June 1, 2020, the patient presented to our hospital. Physical examination revealed a body mass index of 33.96 kg/m² and blood pressure of 140/100 mmHg, with no other significant abnormalities. To facilitate secondary hypertension screening, his antihypertensive regimen was modified to sustained-release nifedipine (30 mg twice daily), with discontinuation of irbesartan, hydrochlorothiazide, and metoprolol tartrate. However, on June 8, 2020, the patient self-administered bisoprolol (5 mg once daily) without medical consultation, citing inadequate blood pressure control. Laboratory tests on June 16, 2020, revealed a plasma renin activity of 0.10 ng/ml/h (reference range: 0.93–6.56 ng/ml/h), plasma aldosterone concentration of 19.82 ng/dL (reference range: 9.8–27.5 ng/dL), an ARR of 198.20 ng/dL: ng/mL·h, and serum potassium level of 3.91 mmol/L. Given the potential for bisoprolol to induce falsely elevated ARR results, his treatment regimen was revised on June 17, 2020, to sustained-release nifedipine (30 mg twice daily) and terazosin (2 mg once daily), with bisoprolol discontinued. Blood pressure monitoring subsequently showed levels ranging between 150–160/95–105 mmHg. Follow-up laboratory tests on July 17, 2020, demonstrated a plasma renin activity of 1.90 ng/ml/h, plasma aldosterone concentration of 24.82 ng/dL, an ARR of 9.07 ng/dL: ng/mL·h, and a serum potassium level of 3.91 mmol/L. Repeat testing on July 24, 2020, showed a plasma renin activity of 3.16 ng/ml/h, plasma aldosterone concentration of 28.67 ng/dL, an ARR of 13.06 ng/dL: ng/mL·h, and a serum potassium level of 3.96 mmol/L. Adrenocorticotropic hormone: 27.81 ng/L (8:00 a.m.), Cortisol: 285 nmol/L (8:00 a.m.). Adrenal magnetic resonance imaging showed no abnormalities in the size, shape, or signal of the bilateral adrenal glands and no abnormal enhancement foci. Comprehensive evaluations, including catecholamines, dopamine, epinephrine, thyroid hormone, alanine aminotransferase, aspartate aminotransferase, urea, creatinine, carotid and renal artery ultrasound, revealed no significant abnormalities. Polysomnography confirmed moderate obstructive sleep apnea-hypopnea syndrome. Additionally, the patient presented with hyperlipidemia, diabetes mellitus, hyperuricemia, hyperinsulinemia, cardiac structural abnormalities, and proteinuria (Table 1). Given the patient’s early-onset hypertension and positive family history, whole exome gene analysis was conducted in July 2020, identifying a variant of uncertain significance in the *CACNA1H* gene, which is associated with familial hyperaldosteronism type IV. The detected heterozygous mutation was as follows: nucleotide change c.3988G > A, amino acid change p.V1330I, and chromosomal location chr16:1260601 (Fig. 1). The variant is located at chromosome 16:1260601, in the 20^{th} exon of transcript NM_021098. With a frequency of 0.00185 in East Asians from the Genome Aggregation Database (gnomAD), it has a REVEL score of 0.290. Neither HGMD (Human Gene Mutation Database) nor ClinVar has reported it, nor have other studies. Per ACMG (American College of Medical Genetics and Genomics) guidelines, it’s rated as a variant of uncertain significance. Sanger sequencing confirmed the presence of the same *CACNA1H* mutation in the patient’s mother. Pedigree analysis demonstrated an inheritance pattern consistent with autosomal transmission (Fig. 2). On August 16, 2020, the patient was advised to follow the Dietary Approaches to Stop Hypertension (DASH) diet and engage in regular aerobic exercise. The patient declined treatment with spironolactone and was subsequently managed with sustained-release nifedipine (30 mg twice daily) and sacubitril/valsartan (200 mg twice daily). During the four-year follow-up, the patient’s blood pressure remained within 120–140/70–90 mmHg, with improved metabolic parameters (Table 1) and stable serum potassium levels (Fig. 3, detailing the treatment course).

**Table 1:** Changes in metabolic characteristics of the patient

Characteristics	Baseline	4 years follow-up	Reference
Blood pressure(mmHg)	140/100	125/83	
Height(cm)	175	175	
Weight(kg)	104	91	
Body mass index(kg/m^2^)	33.96	29.71	18.50-23.99
Triglyceride(mmol/L)	3.30	1.40	0.29–1.83
Total cholesterol(mmol/L)	3.64	4.32	2.80–1.70
High density lipoprotein cholesterol(mmol/L)	0.84	0.83	> 0.90
Low density lipoprotein cholesterin(mmol/L)	1.85	3.00	< 4.00
Uric acid(umol/L)	433	390	240–490
Oral glucose tolerance test			
Fasting plasma glucose(mmol/L)	7.89	6.34	3.90–5.90
2-Hour plasma glucose(mmol/L)	8.89	4.93	3.30–7.80
Insulin release test			
fasting insulin(uU/ml)	25.50	20.93	3.30–7.80
2-Hour postprandial insulin(uU/ml)	205.0	164.55	3.0–60.00
Glycated hemoglobin(%)	6.60	5.53	4.00-6.50
Urine albumin-to-creatinine ratio(mg/mmol)	30	10	< 30
Ultrasonic cardiogram			
Left atrium(mm)	42	29	< 30
Interventricular septum(mm)	14	11	6–12
Ascending aorta(mm)	37	36	20–35

Further review of the patient’s maternal medical history revealed that she had been diagnosed with hypertension at the age of 44. On physical examination, her body mass index was 24.97 kg/m² and her blood pressure was 131/71 mmHg, with no other significant abnormalities detected. She had been receiving oral amlodipine besylate (5 mg once daily) since 1999, maintaining blood pressure within the range of 120–140/70–90 mmHg. Laboratory investigations, including complete blood count, urinalysis, alanine aminotransferase, aspartate aminotransferase, urea, creatinine, uric acid, serum potassium, and blood lipids profiles, revealed no significant abnormalities. Carotid ultrasound demonstrated mild intima-media thickening of both carotid arteries, with atherosclerotic plaque formation in the left carotid sinus. Echocardiography revealed left atrial enlargement, mild mitral regurgitation, mild aortic valve calcification, and a small pericardial effusion. The patient’s mother considered her current blood pressure control satisfactory and declined further tests evaluations, including adrenal hormone testing and adrenal magnetic resonance imaging.

## Discussion and conclusions

Our patient, diagnosed with early-onset hypertension, exhibited poor blood pressure control despite treatment with four antihypertensive agents, including a diuretic. He also presented with multiple metabolic risk factors, including obesity, hyperlipidemia, hyperuricemia, hyperglycemia, and insulin resistance, as well as renal and cardiac target organ damage. Further evaluation for secondary hypertension revealed elevated plasma aldosterone levels; however, The ARR and serum potassium levels were within the normal range. These findings were inconsistent with the typical presentation of primary aldosteronism. Since the both tests showed an ARR below 20 ng/dL: ng/mL·h, further confirmatory tests, such as the saline infusion test or captopril challenge test, were deemed unnecessary according to the diagnostic and treatment protocol outlined in the Expert Consensus on the Diagnosis and Treatment of Primary Aldosteronism [15]. Given the patient’s early onset of hypertension and significant family history, genetic testing was recommended. Genetic analysis identified a heterozygous variant of uncertain significance in the T-type voltage-gated calcium channel subunit alpha-1 H (*CACNA1H*) gene. Sanger sequencing of family members confirmed an autosomal dominant inheritance pattern. The patient exhibited features suggestive of increased aldosterone secretion, potentially due to enhanced activity of the voltage-gated calcium channel (CaV3.2) encoded by CACNA1H. Based on these findings, the patient presents with early-onset hypertension and harbors a CACNA1H variant of uncertain significance.

Familial hyperaldosteronism is classified into four types: type I, type II, type III, and type IV. Affected individuals typically present with clinical features of primary aldosteronism, including dysregulated aldosterone secretion, hypertension, and/or hypokalemia. This condition is characterized by early onset, significant target organ damage, high cardiovascular mortality, and treatment-resistant hypertension [14]. FH-IV was first described in 2015 and is attributed to pathogenic heterozygous mutations in the *CACNA1H* gene, which encodes the CaV3.2 channel [14, 16]. CaV3.2 serves as the primary pathway for calcium ion influx in zona glomerulosa cells. These mutations result in a gain-of-function effect, impairing channel inactivation and shifting activation to more hyperpolarized potentials [16, 17]. Consequently, the increased calcium influx elevated intracellular calcium levels, stimulating increased aldosterone production. This leads to renin-angiotensin system suppression, sodium retention, enhanced potassium excretion, expanded blood volume, and ultimately, hypertension and hypokalemia [18, 19].

A particularly perplexing aspect of this case is the elevated plasma aldosterone levels despite normal ARR and serum potassium levels. A review of the literature suggests two potential explanations for this phenomenon. First, this may be attributed to the incomplete penetrance of *CACNA1H* mutations associated with FH-IV, resulting in significant variability in clinical presentation among affected individuals [11, 14]. Seidel et al. and Geller et al. suggest that dietary salt intake may influence the incomplete penetrance of *CACNA1H* mutations by altering renin-angiotensin system activity, which subsequently affects mineralocorticoid receptor signaling and plasma aldosterone levels [19, 20]. Additionally, cis- or trans-acting genetic modifiers and somatic mosaicism may contribute to this cariability, as both mechanisms can lead to loss-of-function mutations in adrenal cells, thereby influencing aldosterone regulation [21]. Second, this finding may be related to suboptimal blood pressure control, which precluded the complete withdrawal of antihypertensive medications. Although current guidelines and expert consensus suggest that ARR assessment can be performed without full discontinuation of antihypertensive agents, there remains no standardized threshold for ARR interpretation under these conditions [22, 23]. Given that different classes of antihypertensive medications exert variable effects on plasma renin and aldosterone levels, their influence on ARR values introduces additional complexity in the diagnostic evaluation [24]. Current evidence suggests that hydrochlorothiazide and irbesartan may elevate renin levels, potentially resulting in false-negative ARR results, whereas β-blockers may suppress renin levels, leading to false-positive ARR results. In contrast, nifedipine has a relatively minor influence on ARR, possibly causing a slight reduction in aldosterone levels [25]. In our patient, following the discontinuation of all other antihypertensive agents for one month, while maintaining sustained-release nifedipine (30 mg twice daily) and terazosin (2 mg once daily), ARR remained within the normal range. According to the the Expert Consensus on the Diagnosis and Treatment of Primary Aldosteronism [15], confirmatory testing, such as the saline infusion test or captopril challenge test, was not conducted. However, given the patient’s early-onset hypertension and significant family history, genetic testing was recommended. Following the genetic test results, it was determined that the saline infusion test could still provide valid diagnostic information if aldosterone levels remained elevated post-infusion, despite the potential suppression caused by nifedipine. However, the patient declined to discontinue or switch antihypertensive medications due to concerns regarding inadequate blood pressure control. In light of ethical considerations, the patient’s refusal, and the fact that the primary management of FH-IV primarily involves antihypertensive therapy adjustments, we opted not to perform the saline infusion and captopril challenge tests. Upon retrospective analysis, another potential factor affecting ARR results was suboptimal blood pressure control after modification of the antihypertensive regimen. Elevated blood pressure may lead to increased vascular resistance and secondary renin elevation, which could influence ARR interpretation.

Furthermore, the potential impact of isolated aldosterone elevation in this patient should not be overlooked. Notably, elevated plasma aldosterone levels can activate mineralocorticoid receptors across various tissue types, contributing to significant pathophysiological changes. This activation plays a crucial role in cardiac structural remodeling, myocardial hypertrophy, fibrosis, diastolic and systolic function, impaired coronary vascular reactivity, atrial fibrillation induction, inflammatory cell migration, renal fibrosis, albuminuria, and insulin resistance. Consequently, elevated aldosterone levels may independently increase the risks associated with hypertension, obesity, metabolic syndrome, obstructive sleep apnea-hypoventilation syndrome, target organ damage, and related complications [25].

This study has several limitations. First, relying solely on a single aldosterone measurement is inadequate for diagnosing primary aldosteronism, as it cannot differentiate between primary and secondary hyperaldosteronism. The absence of an elevated ARR in this case reduced the diagnostic value of aldosterone measurement, leading to the initial exclusion of primary aldosteronism. As a result, confirmatory tests, such as the saline suppression test or captopril challenge test, were not performed, limiting diagnostic certainty. Second, while genetic testing identified a *CACNA1H* gene mutation, further confirmatory tests for aldosterone secretion could have strengthened the diagnosis. However, due to the patient’s refusal and ethical concerns, these tests were not conducted. Third, This study lacks functional validation of the pathogenicity of the CACNA1H p.V1330I variant. Although it resides in a highly conserved region and co-segregates with early-onset hypertension in the family [26, 27], its classification as a variant of uncertain significance (VUS) in ClinVar—together with the conservative valine-to-isoleucine substitution—weakens any causal inference. Consequently, our findings remain hypothesis-generating, suggesting that the variant may act as a susceptibility modifier rather than a definitive monogenic driver [28]. In addition, several potential confounding factors should be considered, including obesity, metabolic syndrome, and untreated obstructive sleep apnoea. In the review by Wang et al., [29] a bidirectional relationship between obstructive sleep apnoea and aldosterone excess has been demonstrated, whereby obstructive sleep apnoea may increase aldosterone levels, and elevated aldosterone may, in turn, exacerbate obstructive sleep apnoea. This interaction has been implicated in the development of hypertension, particularly resistant hypertension. In the present case, blood pressure improved following weight reduction. therefore, the contribution of metabolic improvement to blood pressure control cannot be excluded. This constitutes an important limitation of the study.

In summary, with the increasing prevalence of early-onset hypertension, there is an urgent need to enhance screening for secondary hypertension. In patients with refractory hypertension, elevated plasma aldosterone levels, and a family history of hypertension, clinicians should consider the possibility of monogenic forms of hypertension, such as familial hyperaldosteronism. Reliance solely on conventional biochemical markers, such as the ARR and serum potassium levels, may lead to missed diagnoses during initial screening, potentially resulting in more severe target organ damage and complications over time. Therefore, in addition to routine biochemical assessments, genetic testing should be considered as a complementary diagnostic tool for patients with early-onset hypertension and a family history of hypertension [11].

## Data Availability

The datasets generated and/or analysed during the current study are available in the China National Center for Bioinformation repository (GSA: HRA010310) that are publicly accessible at [ https://ngdc.cncb.ac.cn/gsa ]( https:/ngdc.cncb.ac.cn/gsa ).

## Abbreviations

ARR: Aldosterone-to-renin ratio
FH-IV: Familial Hyperaldosteronism Type IV
CaV3.2: Voltage-gated calcium channel
PRA: Plasma renin activity
PAC: Plasma aldosterone concentration
gnomAD: Genome Aggregation Database
REVEL: Ranking and Evaluation of the pathogenicity of variants by Ensemble Learning
HGMD: Human Gene Mutation Database
ACMG: American College of Medical Genetics and Genomics
